# Comparison of three electrosurgical modes for endoscopic mucosal resection of 10- to 20-mm colorectal polyps: Randomized controlled trial

**DOI:** 10.1055/a-2663-6177

**Published:** 2025-08-28

**Authors:** Su Luo, Feng Xiong, Sheng-gang Zhan, Zhenglei Xu, Ding-guo Zhang, Ting-ting Liu, Ying-xue Li, Cheng Wei, Ben-hua Wu, Yi-teng Meng, Rui-yue Shi, Jun Yao, Li-sheng Wang, De-feng Li

**Affiliations:** 1 12387 Department of Gastroenterology, Shenzhen People's Hospital, Shenzhen, China

**Keywords:** Endoscopy Lower GI Tract, Polyps / adenomas / ..., Endoscopic resection (polypectomy, ESD, EMRc, ...)

## Abstract

**Background and study aims:**

Endocut Q (effect 2, effect 3 and effect 4) commonly is used for endoscopic mucosal resection (EMR) when removing colorectal polyps. However, there is debate over the type of electrosurgical setting of Endocut Q being recommended in clinical practice. We performed a randomized controlled trial to assess effectiveness and safety of three effects with EMR for removal of non-pedunculated 10- to 20-mm colorectal polyps.

**Patients and methods:**

Patients with non-pedunculate colorectal polyps undergoing EMR were randomly allocated into effect 2, effect 3, and effect 4 groups. The primary outcome was rates of intra-procedural bleeding. Secondary outcomes were rates of post-procedural bleeding, perforation, complete resection, en bloc resection, R0 resection, and residual polyps.

**Results:**

A total of 2637 eligible patients were included in the study and randomly assigned into the effect 2, effect 3, or effect 4 group. There were no significant differences among the three groups in baseline characteristics ( *P* > 0.05). In addition, no significant differences were observed in rates of post-procedural bleeding, perforation, complete resection, en bloc resection, R0 resection, residual polyps, or post-polypectomy syndrome ( *P* > 0.05). However, the rate of intra-procedural bleeding was significantly lower in the effect 2 group than in the effect 3 and effect 4 groups (4.0% vs. 12.2% vs. 12.7%, *P* < 0.01).

**Conclusions:**

Endocut Q (effect 2, effect 3 and effect 4) was effective and safe for removing 10- to 20-mm non-pedunculated colorectal polyps. However, effect 2 may be superior to effect 3 and effect 4 in reducing intra-procedural bleeding.

## Introduction

Endoscopic mucosal resection (EMR) has been the mainstay modality for safe and effective removal of colorectal polyps greater than 10 mm in size, decreasing colorectal cancer related incidence and mortality 1 2 3 . Standard EMR technique requires injection of a liquid solution into the submucosal layer, which lifts the lesions, which are then removed using a hot snare 4 . Thermal injury is a potential risk associated with post-EMR complications, which may include bleeding and perforation 4 5 . However, studies focusing on effects of the electrosurgical settings in relation to post-EMR complications when using EMR to remove colorectal polyps are lacking.

The underlying mechanism of electrosurgery is transformation of energy from the frequency of an electric current into heat, which is then utilized to cut and coagulate tissue 1 6 . Electrosurgical snare resection is widely used to remove large colorectal polyps. However, there is no accepted standard, resulting in various currents and settings being used in clinical practice. The decision behind which current to use has been reported to be primarily based on endoscopist preference. For example, an equal proportion of endoscopists use a type of blended current (46%) and coagulation current (46%) for polypectomy, whereas a smaller proportion of endoscopists use a type of pure cut current (3%) 7 . In 2017, the European Society of Gastrointestinal Endoscopy (ESGE) guideline recommended use of microprocessor-controlled current when using EMR to remove colorectal polyps and recommended against use of low-power coagulation current because of its association with an increased risk of postprocedural bleeding 1 . Pohl H et al reported that there were no significant differences in serious adverse events (AEs), complete resection rate, or polyp recurrence when comparing Endocut Q (a blended current) with coagulation current 8 .

Endocut Q has been characterized as an alternative cut-coagulation current delivered by an electrosurgical unit, which was commonly used for removal of colorectal polyps 6 8 9 . Rates of severe AEs, complete resection, and intraprocedural bleeding were reported to be 7.2%, 96%, and 17% respectively, when Endocut Q (effect 2, duration 1, interval 4) was used to resect colorectal polyps 8 . Endocut Q includes the following effects—effect 2, effect 3, and effect 4—which are commonly applied in clinical practice. However, the optimal effect for EMR when removing colorectal polyps remains unknown. Therefore, in this study, effectiveness and safety of the different effects in the removal of colorectal polyps 10 to 20 mm in size was evaluated.

## Patients and methods

### Study design

A single-center prospective randomized controlled trial was performed at the Shenzhen People's Hospital from February 2020 to April 19, 2025. The study protocol was supported by the Human Ethics Committees of Shenzhen People's Hospital and was conducted according to the Declaration of Helsinki. All participating patients gave informed consent prior to their enrollment. The study was registered at the Chinese clinical trial registry (No. ChiCTR2100048087).

### Study population

Inclusion criteria included that all eligible patients scheduled an EMR to treat non-pedunculated colorectal polyps 10 to 20 mm in size at Shenzhen People's Hospital. For all enrolled patients, only one lesion was resected at the time of the study procedure. Patients who received antithrombotic agents were also required to abstain from them for 7 days, according to professional guidelines 10 . Quality of bowel preparation was evaluated with the Boston Bowel Preparation Scale.

Exclusion criteria included any patients with complications such as severe cardiac, renal and respiratory disease, inflammatory bowel disease, and coagulopathy (international normalized ratio > 1.5, platelets < 50×10 ^9^ /L).

### Randomization and blinding

Eligible patients were randomly assigned to the effect 2, effect 3, or effect 4 group through a computer-generated random number list. Information regarding the randomly assigned effect group was only provided immediately prior to the start of the endoscopic resection procedure.

### EMR procedure

**Fig. 1:**
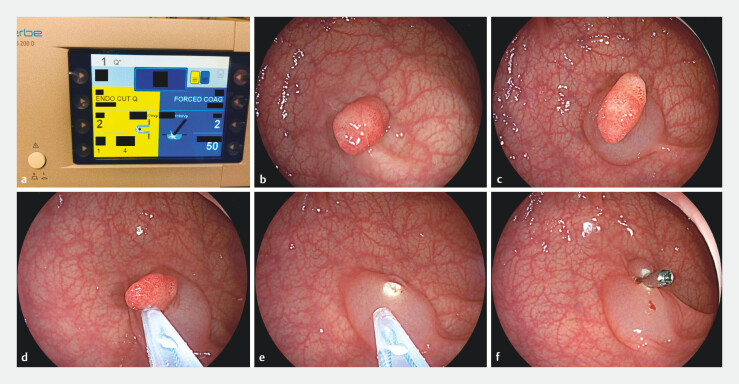
The procedure of EMR.

All eligible patients were administered SIM (200 mg) (Berlin-Chemie AG, Berlin, Germany) in addition to a split-dose 2L polyethylene glycol (Shenzhen Wan he Pharmaceutical Co. Ltd, Shenzhen, China) for bowel preparation, as previously described 11 . All eligible patients received sedation along with intravenous midazolam 5 mg and pethidine 50 mg, as previously described 11 . The brief steps of EMR were as follows: 1) An indigo carmine saline solution was injected into submucosa to lift the colorectal polyps; 2) polyps were removed using an electrosurgical snare (MTN-PFS-E-24/23, Micro-Tech, Nanjing, China) and one of the following settings was applied: effect 2, effect 3 and effect 4 (VIO300D, Erbe, German). Characteristics of the snare used were braided, 24 mm as circle diameter, 0.42 mm as wire diameter. A duration of 1 and interval of 4 were used as unchanged parameters of the Endocut Q settings used; and 3) All cutting was completed within 1 second. Defects were completely closed without obvious defects by the endoscopic clips (Micro-Tech, Nanjing, China). Eight endoscopists with same endoscope technical level performed the research procedure ( Fig. 1 ). All the endoscopists had at least 3 years of operation experience and more than 500 patients of EMR experience. They all had sufficient experience performing EMR with different effects in their routine clinical practice. The patients restarted any antithrombotic agents and followed a restricted diet after the EMR procedure, based on the discretion of the treating endoscopists.

### Outcomes

The primary outcome was the rate of intra-procedural bleeding. Secondary outcomes included intra-procedural perforation, post-procedural AEs, rates of complete resection (based on endoscopic assessment), en bloc resection (based on endoscopic assessment), R0 resection (based on histopathologic assessment), residual polyps, and post-polypectomy syndrome, which were defined as previously described 12 .

Intra-procedural AEs were defined as those occurring during the procedure, which included intra-procedural bleeding and intra-procedural perforation. Intra-procedural bleeding was defined as bleeding during the procedure that could not be stopped without hemostatic intervention. Post-procedural AEs were defined as those occurring within 30 days after the procedure, which included post-procedural bleeding and post-procedural perforation. Characteristics of intra-procedural and post-procedural AEs were identified and the rate of AEs was defined as the number of AEs per patient intra-procedure and post-procedure as previously described 12 .

In this study, a separate comparison was performed of intra-procedure and post-procedure events among these groups. If one patient experienced intra-procedural and post-procedural bleeding in the effect 2 group (100), we calculated that the intra-procedural bleeding rate was 1/100, and the post-procedural bleeding rate was 1/100, whereas we did not calculate the total AE rate as 2/100. If one patient experienced post-procedural bleeding and post-procedural perforation simultaneously in the effect 2 group (100), we calculated that the post-procedural bleeding rate was 1/100 and the post-procedural perforation rate was 1/100, whereas we also did not calculate the total AE rate as 2/100.

### Sample size calculation

Sample size needed to determine statistically significant differences was calculated based on a previous study regarding intra-procedure bleeding events using Endocut Q as compared with forced coagulation currents (17% vs. 11%) 8 . A minimum sample size of 865 was determined to be required, according to α of 0.01, β of 0.1, and a dropout rate of 5% using PASS software.

### Statistical analysis

Continuous variables (ages, body mass index, polyp size, procedure duration, and number of hemoclips) were presented as mean ± standard deviation for normal distributed variables, whereas the median (interquartile range [IQR]) was presented for non-normally distributed variables. Categorical variables were presented as counts and frequency (%). A comparison of means for these three groups was performed using ANOVA. For categorical variables, Chi-squared test, Fisher’s exact test, and the Bonferroni method χ2 test were performed where appropriate. A two-sided *P* < 0.05 was considered statistically significant. All analyses were performed using the SPSS 23.0 software package (SPSS Company, Chicago, Illinois, United States).

## Results

### Baseline characteristics

**Fig. 2:**
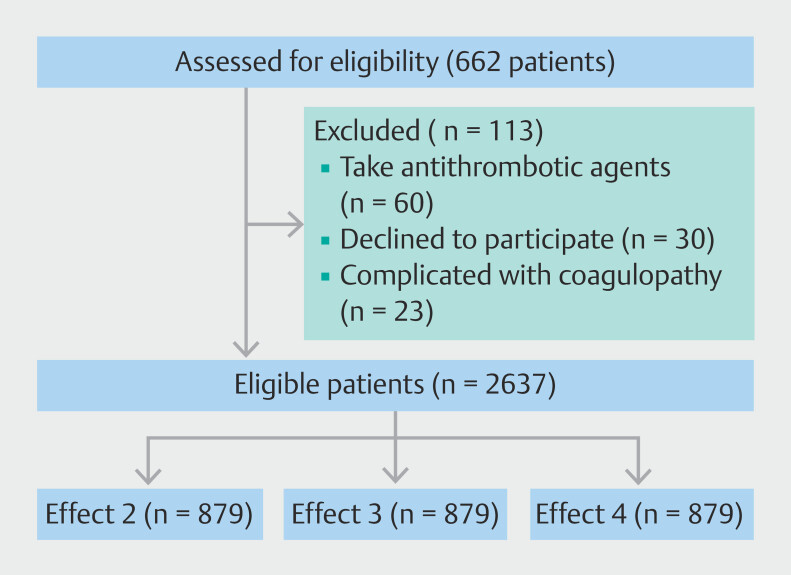
Flow chart of grouping process.

A total of 2750 patients were eligible to enroll and 113 eligible patients were excluded because 60 of them were taking antithrombotic agents, 30 declined to participate, and 23 had coagulopathy complications. Therefore, a total of 2637 patients were enrolled and randomly assigned to the effect 2, effect 3, or effect 4 group ( Fig. 2 ). There were no significant differences in baseline characteristics of the patients in regard to their gender, age, polyp location, procedure duration, number of hemoclips, polyp histology, or polyp histology ( Table 1 ). Every patient received a follow-up telephone call within 30 days after their EMR procedure. A total of 63 patients (27 patients in the effect 2 group, 17 patients in the effect 3 group, and 19 patients in the effect 4 group) did not receive a follow-up surveillance colonoscopy at 6 months after their EMR procedure.

**Table:** **Table 1** Baseline characteristics.

	**Effect 2**	**Effect 3**	**Effect 4**	***P* value **
Gender, n (%)				
Male	518 (58.9%)	492 (56%)	514 (58.5%)	0.40*
Female	361 (41.1%)	387 (44%)	365 (41.5%)	
Age, (years)	57 (33–81)	57.5 (35–80)	58 (34–82)	0.76†
BMI, (kg/m2)	24.1±2.4	23.9±2.32	23.8±2.42	0.58†
Antithrombotic agents, n (%)				
Yes	45 (5.1%)	50 (5.7%)	49 (5.6%)	0.86*
No	834 (94.9%)	829 (94.3%)	830 (94.4%)	
Bowel preparation, n (%)				
Excellent	810 (92.2%)	802 (91.2%)	795 (90.4%)	0.73*
Good	65 (7.4%)	72 (8.2%)	77 (8.8%)	
Fair	4 (0.4%)	5 (0.6%)	7 (0.8%)	
Polyp size, (mm)	15 (10–20)	14.5 (10–19)	14 (10–18)	0.22†
Polyp location, n (%)				
Right side colon	169 (19.2%)	172 (19.6%)	184 (20.9%)	0.71*
Transverse colon	201 (22.9%)	182 (20.7%)	192 (21.8%)	
Left colon	509 (57.9%)	525 (59.7%)	503 (57.3%)	
Procedure duration, (min)	12 (4–20)	12.5 (4–19)	13 (2–18)	0.31 ^‡^
Number of hemoclips, (n)	4.5 (1–10)	4 (1–9)	4 (1–9)	0.22
Histology, n (%)				
Tubular adenoma	692 (78.7%)	690 (78.5%)	671 (76.3%)	0.75 ^*^
Tubulovillous or villous adenoma	126 (14.3%)	118 (13.4%)	127 (14.4%)	
Serrated lesion	45 (5.1%)	49 (5.6%)	58 (6.6%)	
High-grade dysplasia	14 (1.6%)	21 (2.4%)	22 (2.5%)	
BMI, body mass index; left side colon, descending, sigmoid colon, and rectum; right side colon, cecum and ascending colon; transverse colon, hepatic and splenic flexures. ^*^ Bonferroni χ2 test. ^†^ Student’s t-test. ^‡^ Mann–Whitney test.				

### Outcomes

Rates of intra-procedure bleeding were 4.0% (35/879), 12.2% (107/879), and 12.7% (112/879) in the effect 2, effect 3, and effect 4 groups, respectively ( Table 2 ). Therefore, the rate of intra-procedure bleeding in the effect 2 group was determined to be significantly lower than that in the effect 3 and 4 groups ( *P* < 0.01) ( Table 2 ). Rates of post-procedure bleeding were 0.2% (2/879), 0.5% (4/879), and 0.3% (3/879) in the effect 2, effect 3, and effect 4 groups, respectively ( Table 2 ). Of note, there was no significant difference in the rate of post-procedure bleeding among the three groups ( *P* = 1) ( Table 2 ). In addition, no patient experienced intra-procedure or post-procedure perforation in any of the three groups ( Table 2 ). The patients that developed intra-procedure bleeding were treated using hemoclips and did not require a blood transfusion or surgery. Meanwhile, the patients that experienced post-procedural bleeding underwent repeat colonoscopy, had successfully bleeding control, and did not require hospitalization.

**Table:** **Table 2** Outcomes of bleeding and perforation.

	**Effect 2**	**Effect 3**	**Effect 4**	***P* value **
Intra-procedural bleeding, n (%)				
Yes	35 (4.0%)	107 (12.2%)	112 (12.7%)	< 0.01 ^*^
No	844 (96.0%)	772 (93.6%)	767 (93.6%)	
Post-procedural bleeding, n (%)				
Yes	2 (0.2%)	4 (0.5%)	3 (0.3%)	1 ^†^
No	877 (99.8%)	875 (99.5%)	876 (99.7%)	
Intra-procedural perforation, n (%)				
Yes	0	0	0	1 ^†^
No	879	879	879	
Post-procedural perforation, n (%)				
Yes	0	0	0	1 ^†^
No	879	879	879	
^*^ Bonferroni χ2 test. ^†^ Fisher’s exact test.				

Rates of complete resection were comparable among the effect 2, effect 3, and effect 4 groups (100% vs. 100% vs. 100%, respectively) ( Table 3 ). Similarly, there was no significant difference in the rate of en bloc resection among the three groups (100% vs. 100% vs. 100%, respectively) (Table 3). In addition, rates of R0 resection among the three groups were found to be comparable (98.6% [867/879] vs. 98.7% [868/879] vs. 98.3% [864/879], respectively) ( *P* = 0.71) ( Table 3 ). Although the rate of residual polyp was slightly lower in effect 3 group (0.7% [6/862]) than in the effect 2 and effect 4 groups (0.8% [7/852] and 1.3% [11/860]), there was no significant difference among the three groups at 6-month follow-up surveillance colonoscopy ( *P* = 0.42) ( Table 3 ). Moreover, there was no significant difference in the rate of post-polypectomy syndrome among the three groups (2.6% [23/879] vs. 3.0% [26/879] vs. 3.5% [31/879], respectively) ( *P* = 0.54) ( Table 3 ).

**Table:** **Table 3** Outcomes of complete resection.

	**Effect 2**	**Effect 3**	**Effect 4**	***P* value **
Complete resection, n (%)				
Yes	879 (100%)	879 (100%)	879 (100%)	1 ^*^
No	0	0	0	
En bloc resection, n (%)				
Yes	879 (100%)	879 (100%)	879 (100%)	1 ^*^
No	0	0	0	
R0 resection, n (%)				
Yes	867 (98.6%)	868 (98.7%)	864 (98.3%)	071 ^†^
No	12 (1.4%)	11 (1.3%)	15 (1.7%)	
Residual polyp, n (%)				
Yes	7 (0.8%)	6 (0.7%)	11 (1.3%)	0.42 ^†^
No	845 (99.2%)	856 (99.3%)	849 (98.7%)	
Post-polypectomy syndrome, n (%)				
Yes	23 (2.6%)	26 (3.0%)	31 (3.5%)	0.54 ^†^
No	856 (97.4%)	853 (97.0%)	848 (96.5%)	
^*^ Fisher’s exact test. ^†^ Bonferroni χ2 test.				

## Discussion

This is the first randomized trial that compared the effectiveness and safety of three commonly used electrosurgical settings (effect 2 vs. effect 3 vs. effect 4) for removal of colorectal polyps. The results of this study showed no significant differences among the three groups in regard to intra-procedure perforation, post-procedure perforation, complete resection, en bloc resection, R0 resection, residual polyps, or post-polypectomy syndrome. However, the rate of intra-procedural bleeding was significantly lower in the effect 2 group compared with the effect 3 and 4 groups. Notably, no severe AEs occurred in any of the enrolled patients, highlighting the safety and effectiveness of each of the electrosurgical settings for removal of colorectal polyps.

The substantial aspect of polypectomy is the electrophysiological effect of the current delivered through the snare 1 . The essential underlying differences among effect 2, effect 3, and effect 4 are that the effect 2 is low effect resection, the effect 3 is moderate effect resection, and the effect 4 is high effect resection. However, when the higher effective resection is used, there is a greater risk of intra-procedural bleeding because the cutting pulse is not sufficient to seal the bleeding vessels 8 . Therefore, it is possible to explain why the effect 2 settings were associated with less intra-procedural bleeding for removal of colorectal polyps in the present study. A large randomized trial carried out by Pohl. H et al reported that rates of intra-procedural bleeding, post-procedural bleeding, perforation, and residual polyp were 17.4%, 5.0%, 0.9%, and 45.2% when using Endocut Q (effect 2) to remove colorectal polyps, which was markedly higher than our results 8 . In addition, the rate of complete resection reported in the Pohl. H et al study was slightly lower than what we observed in our study 8 . The primary underlying reasons for this may be as follows: 1) colorectal polyp size was larger in the Pohl. H et al study than the ones in our study; 2) nearly 17% of colorectal polys did not lift completely with the submucosal injection in the Pohl. H et al study; however, all colorectal polyps were lifted with the submucosal injection in our study; and 3) approximately 53.8% of mucosal defects after polypectomy were not closed in the Pohl. H et al study, whereas all the mucosal defects were closed after polypectomy using hemoclips in our study. This is consistent with previous studies, which have reported that colorectal polyp size may be an independent risk factor for complete resection, AEs, and residual polyps when using EMR to remove colorectal polyps 13 14 15 .

In the present study, none of the enrolled patients developed intra-procedural or post-procedural perforation. The main reason may be that a liquid solution injection into the submucosal layer formed a safety cushion and protected deeper layers from thermal injury. Van Hattem WA et al demonstrated that the rate of delayed perforation was less than 0.6% when using EMR to remove large colorectal sessile serrated polyps (≥ 20 mm) 16 , which was slightly higher than our results. A meta-analysis showed that rates of post-procedural bleeding, perforation, and residual polys were 0.6%, 0%, and 1.2% respectively, when EMR was used to remove sessile serrated lesions 10 to 19 mm in size, which is consistent with our results 17 . However, the intra-procedural bleeding rate was 0.6%, which was markedly lower than what was observed in the effect 2, effect 3, and effect 4 groups in the present study (0.6% vs. 4.0% vs. 12.2% vs. 12.7%) 17 . The main reason may be that the total sample size in each group was much larger in the present study compared with the above-mentioned study (879 vs. 563) 17 .

Post-polypectomy syndrome is an uncommon complication of EMR for removal of colon polyps 18 . Post-polypectomy syndrome occurs when electrosurgical current penetrates beyond the mucosa during polypectomy, affecting the muscularis propria and serosa. This creates a transmural thermal injury, which triggers peritoneal inflammation, yet without full-thickness perforation 18 . It has been reported that the rate of post-polypectomy syndrome ranges from 0.2% to 7.6% 19 20 . In this study, incidence of post-polypectomy syndrome was 2.6%, 3.0%, and 3.5% in the effect 2, effect 3, and effect 4 groups, respectively. The rate of post-polypectomy syndrome was slight lower in the effect 2 than in the effect 3 and 4 groups, which may be ascribable to the lower electrosurgical current in the effect 2 group.

Several limitations of the current study should be noted. First, this study was performed in a single center. Second, we have not included colorectal polyps > 20 mm in size. The R0 resection rate was less than 78.8%, whereas the residual rate was as higher as 6% when using EMR to remove non-pedunculated colorectal polyps > 20 mm in size 17 . Therefore, endoscopic submucosal dissection was generally performed to treat non-pedunculated colorectal polyps > 20 mm in size in our clinical center. Third, it has been shown that intervention modalities such as argon plasma coagulation and snare tip soft coagulation ablate the EMR margin and decrease the residual rate 21 22 . However, intervention modalities were not used to ablate the EMR margin in the present study because the residual rate was less than 0.6% when using EMR to remove non-pedunculated colorectal polyps < 20 mm in size 17 . Fourth, experienced endoscopists were utilized in present study; thus, we cannot guarantee that the results would be reproducible in other clinical centers with less experienced endoscopists. Fifth, it is noted that clips were placed on all defects after EMR resection, which could have impacted the rate of post-procedural AEs. Therefore, the rate of post-procedural AEs in this study did not reflect the pure incidence of post-procedural AEs among the effect 2, effect 3, and effect 4 groups.

## Conclusions

In conclusion, the electrosurgical settings of Endocut Q (effect 2, effect 3, and effect 4) were safe and effective for removal of non-pedunculated colorectal polyps 10 to 20 mm in size. However, effect 2 may be superior to both effect 3 and effect 4 for decreasing occurrence of intra-procedural bleeding. All of these results were obtained by experienced endoscopists.
